# Impact of the COVID-19 pandemic and Honey Rose case on hospital attendances of patients suspected to have papilloedema

**DOI:** 10.1038/s41433-022-02310-0

**Published:** 2022-11-28

**Authors:** Catherine McNicholl, Arran Gill, Rhys Harrison, Denize Atan

**Affiliations:** 1 Translational Health Sciences, University of Bristol Medical School, Beacon House, Queen’s Road, Bristol, BS8 1QU UK; 2 Bristol Eye Hospital, University Hospitals Bristol & Weston NHS Foundation Trust, Lower Maudlin Street, Bristol, BS1 2LX UK; 3 University of Southampton Medical School, University Road, Southampton, SO17 1BJ UK

**Keywords:** Health care, Signs and symptoms, Diseases

Papilloedema can be the first sign of life-threatening disease. Its importance was highlighted in 2016 when optometrist, Honey Rose, was convicted of gross negligence manslaughter for not identifying papilloedema in a child who later died [1]. As the detection of papilloedema relies entirely on fundal examination and/or imaging that were relatively contraindicated among non-eyecare clinicians during the COVID-19 pandemic, we were concerned about the impact of the pandemic on hospital attendances of patients with papilloedema.

With National Health Research Authority ethical approval (IRAS: 306282), we extracted anonymised data on new patients attending the Accident & Emergency (A&E) and neuro-ophthalmology services at Bristol Eye Hospital (BEH) from Trust electronic patient records. Our analysis only included adults with suspected papilloedema because of headaches and/or indistinct optic disc margins and excluded patients with other causes of optic disc swelling (e.g., ischaemic optic neuropathy, optic neuritis, giant cell arteritis). Children were excluded because they are normally directed elsewhere within the Trust. Referrals were further classified as “true” or “false”-positives depending on whether papilloedema was confirmed by subsequent investigation.

As expected, fewer patients with suspected papilloedema attended BEH during the first lockdown (23 March to 14 June 2020) than before or afterwards. However, the impact was greatest on the large proportion of false-positive referrals before (78.1%; 118/151), during (64.3%; 36/56), and after lockdown (79.7%; 141/177). Surprisingly, 60.1% (179/295) of false-positives actually had normal optic discs: 55.9% (100/179) of these had headaches but normal eye examinations, 7.3% (13/179) had non-specific visual symptoms; and 36.9% (66/179) were asymptomatic. True-positive cases were diagnosed with idiopathic intracranial hypertension (49%; 44/89), space-occupying lesions (10%; 9/89) and other aetiologies (41%; 39/89) but there was no evidence that any true-positive cases were missed (Fig. 1).

**Fig. 1:**
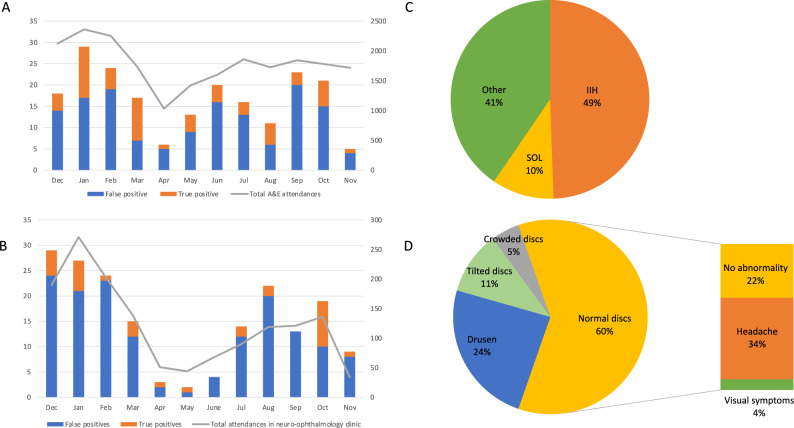
The impact of the COVID-19 pandemic on the hospital attendances of patients suspected to have papilloedema at Bristol Eye Hospital. Total number of attendances in **A** A&E department and **B** neuro-ophthalmology clinics (dark grey lines, right y-axis) plotted against number of true-positive (orange) and false-positive (blue) referrals for suspected papilloedema at Bristol Eye Hospital (left y-axis). Causes of **C** true-positive referrals and **D** false-positive referrals to the A&E and neuro-ophthalmology services before, during and after the first COVID-19 lockdown in England. Bar graph shows the false-positive cases with normal discs, and their percentage breakdown by reason for referral (total = 60%). Abbreviations: IIH Idiopathic intracranial hypertension, SOL space-occupying lesion.

We were interested to know whether the large proportion of false-positive referrals for suspected papilloedema dated back to the widespread media coverage of the Honey Rose case in July 2016, when others had reported increased demand on their NHS services [2]. Indeed, false-positive referrals had jumped from 33.3% in 2015 to 60.9% in 2016 while pressure on the neuro-ophthalmology service had increased by 500–600% (Fig. 2).

**Fig. 2:**
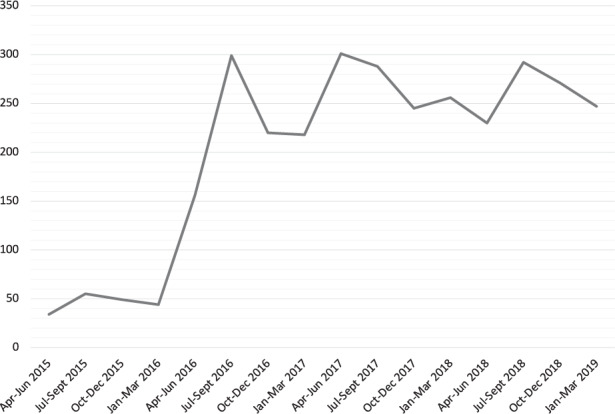
Quarterly number of new hospital attendances within the neuro-ophthalmology service at Bristol Eye Hospital between 2015 to 2019.

With the introduction of optometrist-led community services for minor eye conditions (MECS) and COVID-19 urgent eyecare (CUES), and the decline in ophthalmology teaching at undergraduate level [3], optometrists have become de facto gatekeepers to NHS secondary eyecare services. Yet, concerns about missing sight- or life-threatening diagnoses, like papilloedema, has meant referral thresholds have decreased since Honey Rose. Further, the introduction of OCT to many optician practices [4] has increased referrals of patients who don’t need medical treatment, not decreased them [5]. Although patients with suspected papilloedema are proportionately few (~1% A&E and 8–12% neuro-ophthalmology attendances), substantial healthcare costs are accrued by unnecessary hospital appointments, investigations, time off work, longer waiting times, and by increasing personnel/clinic capacity to meet higher service demands. Given the huge backlog in appointments and operations caused by COVID-19, the time is ripe to rethink how primary and secondary eyecare services could work better together to serve the patients who need them most.

## Data Availability

Anonymised data from this study is available on request.
